# Voter Support for Policies Associated With Child Health as National Campaign Priorities

**DOI:** 10.1001/jamahealthforum.2024.3305

**Published:** 2024-09-27

**Authors:** Stephen W. Patrick, Sarah F. Loch, Elizabeth McNeer, Matthew M. Davis

**Affiliations:** 1 Department of Health Policy and Management, Rollins School of Public Health, Emory University, Atlanta, Georgia; 2 Department of Pediatrics, Emory University School of Medicine, Emory University, Atlanta, Georgia; 3 Department of Biostatistics, Vanderbilt University Medical Center, Nashville, Tennessee; 4 Nemours Children’s Health, Wilmington, Delaware

## Abstract

**Importance:**

Policies that are associated with child health are rarely included in platforms of candidates for national political office. Candidates may underrecognize voter support for such priorities or perceive that such policy issues are not sufficiently divisive to appeal to partisan voters. Key policy questions associated with child health may be considered by the next Congress, including the consistency of Medicaid coverage across states and restoring the recently lapsed refundable child tax credit.

**Objective:**

To examine voter support for candidates regarding policies that are associated with child health.

**Design, Setting, and Participants:**

This nationally representative survey of registered US voters 18 years or older was conducted from March to April 2024 and included a survey-based randomized experiment to evaluate the association of message framing with voter support.

**Exposures:**

Messages conveying distinct rationales for Medicaid reform and refundable child tax credit.

**Main Outcomes and Measures:**

Likely or definite support for candidates.

**Results:**

In this sample (unweighted N = 2014; 1015 women [51.0%]), most respondents indicated they would likely or definitely vote for candidates who expressed strong support for all tested policies: extreme risk protection order (79.5%), school threat assessment (73.1%), expanded childcare (69.6%), refundable child tax credit (66.6%), federalization of Medicaid (66.0%), paid parental leave (65.5%), free school meals (65.6%), safe firearm storage and enforcement (62.9%), preventing Medicaid disenrollment for children younger than 6 years (61.9%), universal free preschool (61.6%), and summer nutrition programs (57.9%). More women than men expressed support for all tested policies. Strong majorities of Democrat and Independent voters would support candidates who endorsed child-focused policies; fewer than 50% of Republican voters expressed such support, except for the extreme risk protection order and school threat assessment. Variations in framing language regarding consistent Medicaid coverage across states were not associated with amplified or diminished voter support. Framing the refundable child tax credit as benefiting “hard-working” vs “low-income” families garnered significantly more support among men (67.0% vs 59.0%), privately insured individuals (72.0% vs 64.4%), and Republicans (54.6% vs 43.0%; all *P* < .05).

**Conclusions and Relevance:**

The study results suggest that most voters favor candidates who strongly support policies that are associated with child health. Voter support differs substantively by gender and political party affiliation and may be associated with language choices in messaging about policy change.

## Key Points

**Question:**

Policies that are associated with child health are rarely included among political candidates’ campaign priorities, but would US voters support candidates with a child-focused legislative agenda?

**Findings:**

In this nationally representative survey study of 2014 participants, most registered voters would be likely to vote for candidates who strongly support national funding for multiple different policy issues associated with child health, including equitable Medicaid coverage for children across all states. Voter support was associated with gender, political party, and the presence of children younger than 18 years in the household.

**Meaning:**

The study results suggest that political candidates may underappreciate broad public support for policy priorities that promote child health.

## Teaser

This survey study examines US voter support for candidates regarding policies that are associated with child health.

## Introduction

Despite the fact that children younger than 18 years comprise 22% of the US population, child health priorities are rarely included in the policy platforms of political campaigns for national office.^1,2,3^ Such underrepresentation may result from an underappreciation among candidates of support for child health policy issues among their potential constituents. However, if support for such issues is broad across political parties, candidates who seek to campaign primarily on divisive topics in the current highly partisan political environment^4^ may not prioritize policies that are associated with child health. To our knowledge, these hypotheses have not been previously evaluated.

In the US, 2 federal policy questions have recently emerged that may have the potential to affect children’s health and well-being in the short term and long term. First, in late 2023, the American Academy of Pediatrics proposed foundational changes in Medicaid and the Child Health Insurance Program, which currently provide coverage for approximately one-half of all US children. The proposed modifications would remove state-by-state variation in coverage and benefits through the programs to ensure greater equity in access to health care for children no matter their state of residence; such changes would require Congressional action.^5^ Second, the Expanded Child Tax Credit enacted in 2021 (also called the refundable child tax credit) as part of the American Rescue Plan improved family household incomes so that more than 2 million children were no longer living in poverty.^6^ This tax credit was allowed to lapse in 2022, which was followed by an increase in child poverty rates^7^ that are known to be associated with worse child health outcomes.^8^ Consequently, child health advocates and policymakers have advocated for re-establishing the expanded child tax credit.

Despite their policy relevance for the upcoming 2024 election, data are scarce regarding support among US voters for these specific Medicaid and refundable child tax credit policy options or the broader question of support for candidates whose campaign platforms prioritize child health policy issues. Therefore, we conducted a national study among voters in 2024 to interrogate these issues and inform the electorate and candidates themselves about child health policy in the current political landscape.

## Methods

### Design and Study Population

We conducted a cross-sectional survey and survey-based experiment that was designed to elucidate voting preferences of US adults regarding child policy priorities of candidates running for national office in 2024. We fielded the survey from March 27 through April 12, 2024, using the Ipsos KnowledgePanel, the largest online panel in the US that relies on probability-based sampling methods for recruitment to provide a sampling frame for noninstitutionalized adults in the US that has served as the sampling frame for many peer-reviewed publications regarding public perceptions of health, health care, public health, and health policy.^9,10,11,12,13,14,15^

For this study, we included respondents in KnowledgePanel at least 18 years old who self-reported as registered voters. We oversampled parents with at least 1 child in the household who was younger than 18 years to achieve approximately 50% of the unweighted sample (hereafter, *parent* refers to an individuals who has a child younger than 18 years the in home; *other adult* refers to an individual without a child younger than 18 years in the home). Eligible participants were randomly selected from the standing panel and sent an email notification, and nonresponders were sent subsequent reminders at days 3, 8, and 14 of the fielding period. Households without internet access at the time of recruitment were provided with an internet-enabled tablet. Participants in the panel received nominal periodic incentives to participate in various surveys on multiple topics unrelated to the study described in this article. This study followed the best practices of the American Association for Public Opinion Research. This survey of unidentified persons was considered exempt from human participants review by the Vanderbilt University Medical Center institutional review board, which also waived informed consent.

### Survey Instrument

For this study regarding health and social policies associated with children’s health, we developed an original survey instrument (eAppendix in Supplement 1) to examine registered voter preferences using a series of vignettes and direct questions to measure voter attitudes. In survey-based experiments, respondents are randomly assigned to a vignette that “exposes” them to information and are then asked a uniform set of questions. The random assignment permits researchers to isolate the association of specific aspects of the information exposure with respondents’ answers. This established research approach has been used widely in sociobehavioral and economic research and has served as the basis for published studies regarding child health and health policy.^16,17,18^ For this study, we constructed brief vignettes to test voter preferences regarding Medicaid and the refundable child tax credit. One set of vignettes tested voters’ likelihood of supporting efforts to nationalize Medicaid using 3 message frames: (1) equity in coverage, (2) fairness of coverage, or (3) loss of opportunity for coverage. Another set of vignettes tested voters’ support for reinstating a refundable tax credit based on framing affected parents as (1) “low-income” vs (2) “hard-working.” Framing for the latter vignette was based on recent national media descriptions of the child tax credit.^19,20^

Separate from vignettes, the survey asked respondents about their likelihood of voting for a candidate who stated they would strongly support national funding for various specific policy initiatives that have been proposed as opportunities to protect and promote children’s health: universal preschool for 3-year-old and 4-year-old children^21,22^; expanding childcare access^23^; making school meals free for all children^24,25,26^; establishing a summer nutrition program for children^27,28^; paid parental leave^29,30^; federalizing Medicaid^5,31^; an extreme risk protection order (ERPO) or “red flag law”;^32^ school threat assessment and identification programs to prevent active shooters^33,34^; providing safe firearm storage and requiring safe use^35^; and preventing states from disenrolling children younger than 6 years from Medicaid.^5,36^ The survey was administered in English and Spanish.

To assure that our survey questions were eliciting an array of responses as intended, we pilot-tested the instrument with 25 respondents from the panel. For all questions, the questions yielded a broad distribution of responses across the response choice spectrum, and the instrument was fielded as designed.

### Study Data

Survey weights were designed to provide national estimates using data obtained from the 2023 March Supplement of the Current Population Survey^37^ for all variables, except for language proficiency, which was obtained from the 2022 American Community Survey.^38^ Survey weights were constructed by ranking geodemographic distributions of the parent population (age ≥18 years) who have children aged 0 to 17 years. Once all survey data were collected, design weights were adjusted to account for differential nonresponse. The following demographic data were collected and used in survey weights: respondent self-identified gender (female, male), respondent age (18-29, 30-44, 45-59, and ≥60 years), race and ethnicity (Hispanic, non-Hispanic Black, non-Hispanic multiracial, non-Hispanic other race, and non-Hispanic White), census region (Northeast, Midwest, South, and West), annual household income (<$10 000, $10 000 to<$25 000, $25 000 to<$50 000, $50 000 to<$75 000, $75 000 to<$100 000, $100 000 to<$150 000, and ≥$150 000), home ownership status, household size, metropolitan status (metropolitan, nonmetropolitan), Hispanic origin (Cuban, Mexican, non-Hispanic, Puerto Rican, or other), and language.

### Data Analysis

We conducted and report all analyses using survey weights to yield nationally representative estimates. Demographic characteristics of the respondents were summarized using unweighted frequencies, weighted percentages, and standard errors overall and by parental status. Descriptive statistics were calculated to summarize response frequency. Respondents who refused to answer a question were considered missing and not included in calculating proportions; refusals were less than 1.1% for all questions. We report summary statistics as weighted proportion estimates of a positive response (eg, favor or strongly favor vs other options; likely or definitely vote for vs other options), in most cases with a corresponding 95% CI. The CIs were calculated using logistic regression models. We conducted significance testing using Rao-Scott corrected χ^2^ tests. The significance level was set at α = .05, and all tests were 2-sided. All analyses were conducted using the survey package^39^ in R, version 4.2.1 (R Core Team).

## Results

This survey had a qualification rate of 51%, 64% completion rate,^40^ median completion time of 7 minutes, and total of 2014 responses that were suitable for analyses after 52 cases were dropped for completing the survey in less than 33% of the median interview length (ie, speeding). The unweighted analytic sample included 2014 registered voters, including 1030 parents (51.1%) with children younger than 18 years in the household and 984 other adults (48.9%). After applying survey weights, parents were more likely than other adults to be aged 30 to 44 years (59.9% vs 14.4%), currently married (74.0% vs 49.8%), have private insurance (79.5% vs 60.9%), and have a Bachelor’s degree or higher (50.6% vs 37.8%). They were somewhat less likely to identify as a Democrat (30.4% vs 37.8%) and more likely to identify as an Independent (39.3% vs 33.4%; Table 1).

**Table 1.** Characteristics of Registered US Voter Respondents in Study Sample From March to April 2024

Characteristic	Weighted No. (%) [SE]		
	Parents	Other adults	Total
Age, y			
18-29	79 (9.8) [1.3]	118 (19.9) [1.6]	197 (17.6) [1.3]
30-44	587 (59.9) [1.7]	114 (14.4) [1.3]	701 (24.8) [1.1]
45-59	328 (25.5) [1.4]	227 (21.8) [1.3]	555 (22.6) [1.1]
≥60	36 (4.7) [0.9]	525 (43.9) [1.7]	561 (34.9) [1.3]
Gender			
Female	521 (53.5) [1.8]	494 (50.3) [1.7]	1015 (51.0) [1.4]
Male	509 (46.5) [1.8]	490 (49.7) [1.7]	999 (49.0) [1.4]
Marital status			
Now married	796 (74.0) [1.7]	566 (49.8) [1.7]	1362 (55.4) [1.4]
Unmarried	234 (26.0) [1.7]	418 (50.2) [1.7]	652 (44.6) [1.4]
Urbanicity			
Urban	342 (34.5) [1.7]	334 (35.7) [1.7]	676 (35.4) [1.3]
Rural	198 (20.0) [1.4]	206 (19.7) [1.3]	404 (19.7) [1.1]
Suburban	490 (45.6) [1.7]	444 (44.7) [1.7]	934 (44.9) [1.4]
Insurance			
Private^a^	829 (79.5) [1.5]	589 (60.9) [1.7]	1418 (65.2) [1.3]
Public^b^	149 (14.7) [1.3]	344 (33.4) [1.6]	493 (29.1) [1.3]
Other^c^	7 (0.6) [0.2]	11 (1.3) [0.4]	18 (1.1) [0.3]
None	43 (5.2) [0.9]	34 (4.5) [0.8]	77 (4.6) [0.6]
Education			
≤High school	221 (24.7) [1.6]	311 (34.7) [1.7]	532 (32.4) [1.3]
Some college	243 (24.8) [1.5]	272 (27.5) [1.5]	515 (26.9) [1.2]
≥Bachelor’s degree	566 (50.6) [1.8]	401 (37.8) [1.6]	967 (40.7) [1.3]
Adult health status			
Excellent	105 (10.3) [1.1]	82 (9.1) [1.1]	187 (9.4) [0.8]
Very good	458 (44.2) [1.7]	372 (36.9) [1.6]	830 (38.6) [1.3]
Good	357 (34.8) [1.7]	367 (36.3) [1.6]	724 (35.9) [1.3]
Fair	100 (9.7) [1.1]	139 (15.3) [1.3]	239 (14.0) [1.0]
Poor	10 (0.9) [0.3]	22 (2.4) [0.5]	32 (2.1) [0.4]
Child health status			
Excellent	468 (44.7) [1.7]	NA	468 (44.7) [1.7]
Very good	441 (42.5) [1.7]		441 (42.5) [1.7]
Good	111 (11.9) [1.2]		111 (11.9) [1.2]
Fair	8 (1.0) [0.4]		8 (1.0) [0.4]
Poor	0		0
Political party affiliation			
Republican	323 (30.2) [1.6]	310 (28.8) [1.5]	633 (29.1) [1.2]
Democrat	301 (30.4) [1.6]	345 (37.8) [1.7]	646 (36.1) [1.4]
Independent^d^	404 (39.3) [1.7]	325 (33.4) [1.6]	729 (34.8) [1.3]
Survey language			
English	1002 (96.7) [0.7]	967 (97.7) [0.6]	1969 (97.4) [0.5]
Spanish	28 (3.3) [0.7]	17 (2.3) [0.6]	45 (2.6) [0.5]

Abbreviation: NA, not applicable.

^a^ Includes anyone covered by insurance through a current or former employer or union or insurance purchased directly from an insurance company.

^b^ Includes anyone covered by Medicare, Medicaid, TRICARE, Veteran’s Affairs, US Department of Defense, other military programs, or Indian Health Service and not covered by any of the private insurance categories.

^c^ Includes anyone covered by any other type of health insurance or health coverage plan and not covered by any of the private or public Insurance categories.

^d^ Independent includes respondents who selected Independent or something else.

**Figure 1.**
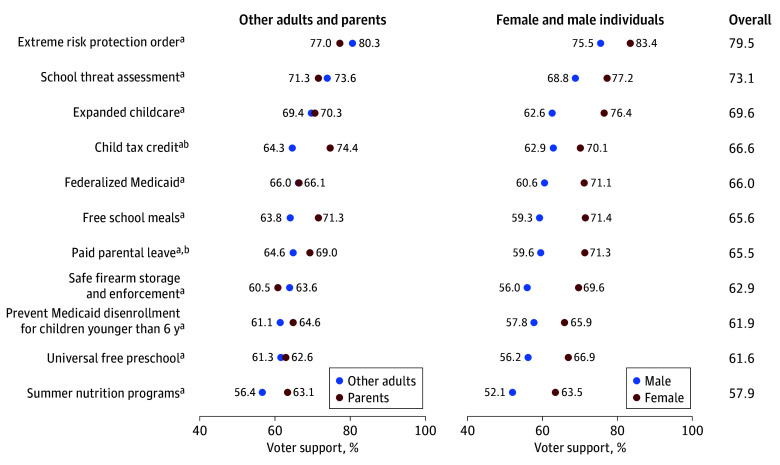
Voter Support for Candidates Who Strongly Support National Funding for Policies Affecting Child Health by Gender and Parental Status of Respondents *Voter support* defined as will likely vote or will definitely vote for a candidate. *Parent* defined as having at least 1 child younger than 18 years in the household. ^a^*P* < .05 for gender. ^b^*P* < .05 for parental status.

Overall, most voters indicated their support (would likely or definitely vote) for candidates who expressed strong support across a range of policies associated with child health, including universal ERPO (79.5%), school threat assessment (73.1%), expanded available childcare (69.6%), refundable child tax credit (66.6%), federalized Medicaid (66.0%), paid parental leave (65.5%), free school meals (65.6%), safe firearm storage and enforcement (62.9%), prevention of Medicaid from disenrolling children younger than 6 years (61.9%), universal free preschool for 3-year-old and 4-year-old children (61.6%), and summer nutrition programs (57.9%; Figure 1).

Voter support for such policies varied substantially by respondent gender, with higher proportions of women than men expressing support for all tested issues. Support for expanding childcare varied most by gender, with 76.4% of women expressing candidate support (95% CI, 73.2%-79.4%) compared with 62.6% of men (95% CI, 58.8%-66.2%). In contrast, support for reinstating the refundable child tax credit varied least by gender, with 70.1% of women expressing candidate support (95% CI, 66.5%-73.5%) compared with 62.9% of men (95% CI, 59.1%-66.6%; Figure 1B). Compared with other adults, parents were significantly more likely to say they would vote for candidates who strongly supported restoring the refundable child tax credit, paid parental leave, and expanding summer nutrition programs. Other adults were more likely than parents to express support for candidates who campaigned on ERPOs (Figure 1A).

**Figure 2.**
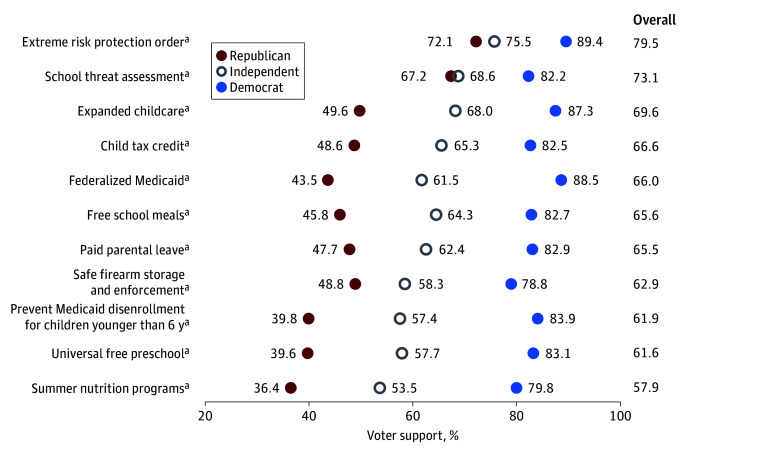
Voter Support for Candidates Who Strongly Support National Funding for Policies Affecting Child Health by Political Party Affiliation *Voter support* defined as will likely or will definitely vote for a candidate. ^a^*P* < .05.

Support for candidates regarding policies associated with child health varied substantially by political party affiliation, with the highest proportion of support among voters who identified as Democrats. For all tested policies, most Democrat and Independent voters expressed support for candidates who supported such policies. Candidates favoring ERPOs had strong support among all voters, with a maximum difference of 17.3 percentage points across party affiliations (Democrats, 89.4% [95% CI, 85.6%-92.3%]; Independents, 75.5% [95% CI, 71.2%-79.3%]; Republicans, 72.1% [95% CI, 67.8%-76.1%]). Support for candidates who endorsed federalizing Medicaid had the largest gap in support by party affiliation of 45 percentage points (Democrats, 88.5% [95% CI, 84.9%-91.2%]; Independents, 61.5% [95% CI, 56.9%-65.8%]; Republicans, 43.5% [95% CI, 38.9%-48.3%]; Figure 2).

**Figure 3.**
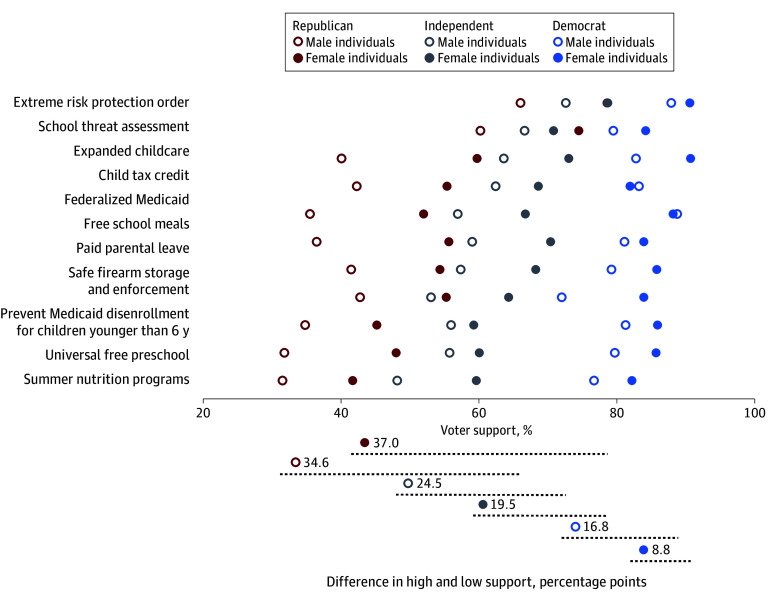
Voter Support for Candidates Who Strongly Support National Funding for Policies Affecting Child Health by Combined Gender and Political Party Affiliation *Voter support* defined as will likely or will definitely vote for a candidate.

Examination of voter preferences across combined gender and political party affiliation subgroups revealed that, on average, a minority of male Republicans said that they would vote for candidates who strongly supported policies associated with child health (mean of 42.0% of voters across all tested policies). Mean levels of voter support were progressively higher among female Republicans (56.3%), male Independents (59.2%), female Independents (67.2%), male Democrats (81.1%), and female Democrats (85.7%). The support of Republican voters differed more broadly than others regarding specific policies. The support of female Republicans varied 37 percentage points from least support to most, followed by male Republicans (34.6 points), male Independents (24.5 points), female Independents (19.5 points), male Democrats (16.8 points), and female Democrats (8.8 points; Figure 3).

### Framing Experiment: Ensuring Consistent Child Coverage Within Medicaid Across All States

To test if different messages were associated with support for consistent Medicaid coverage across states, respondents were randomized to read a vignette that varied in framing language (eAppendix in Supplement 1). Overall, framing Medicaid coverage with different messages was not associated with support for Medicaid reform focused on children. By political party affiliation, framing did not vary support for ensuring consistent Medicaid coverage among self-identified Democrats or Republicans. However, Independents were more likely to support federalizing Medicaid when responding to an equity (71.8%) or loss (72.6%) vignette than they were a fairness vignette (59.1%). Voter support did not differ across message framing within any other demographic group (eTable in Supplement 1).

### Framing Experiment: Restoring the Refundable Child Tax Credit

To test how framing was associated with support for restoring the refundable child tax credit, respondents were randomly assigned vignettes that framed the policy as benefiting “hard-working” vs “low-income” families. Framing the policy as benefiting hard-working families garnered a significantly more supportive response among men (67.0% for hard-working vs 59.0% for low-income), privately insured individuals (72.0% vs 64.4%), and Republicans (54.6% vs 43.0%). No demographic subgroup was significantly more supportive of framing the benefit regarding low-income families than for hard-working families (Table 2). Within the subgroups of respondents assigned to the 2 vignettes, voter support differed significantly by sociodemographic factors, including age, urbanicity, health insurance coverage, and political party affiliation. Among respondents assigned to the low-income vignette, there was also a difference in voter support by respondent gender.

**Table 2.** Voter Support for Restoring the Refundable Child Tax Credit Compared With Message Framing Presented in Randomly Assigned Vignettes

Characteristic	% (95% CI)^a^	
	Hard working (n = 1004)	Low income (n = 1003)
Gender		
Female	71.4 (66.2-76.0)	68.8 (63.6-73.6)
Male	67.0 (61.4-72.2)	59.0 (53.6-64.2)
Age, y		
18-29	66.8 (54.6-77.1)	73.6 (61.9-82.6)
30-44	76.2 (69.7-81.6)	69.0 (61.9-75.3)
45-59	63.8 (56.2-70.8)	52.6 (45.1-60.0)
≥60	69.4 (63.4-74.8)	62.9 (56.8-68.5)
Parental status		
Parent	75.4 (70.8-79.5)	73.4 (69.0-77.4)
Nonparent	67.4 (62.7-71.8)	61.1 (56.5-65.6)
Urbanicity		
Urban	73.8 (67.8-79.1)	66.0 (59.3-72.0)
Suburban	67.3 (61.6-72.6)	66.7 (61.2-71.9)
Rural	64.3 (54.9-72.7)	55.2 (47.1-63.0)
Health status, adult		
Worse (F/P)	67.8 (57.1-76.9)	62.0 (51.4-71.6)
Better (E/VG/G)	69.5 (65.5-73.2)	64.1 (60.1-68.0)
Health status, child		
Worse (G/F/P)	76.5 (61.3-87.0)	69.6 (54.4-81.5)
Better (E/VG)	75.6 (70.7-79.9)	74.0 (69.4-78.1)
Insurance type		
Public	65.8 (58.2-72.6)	63.1 (55.9-69.7)
Private	72.0 (67.6-76.0)	64.4 (59.8-68.8)
Other	80.9 (28.9-97.8)	48.2 (15.0-83.0)
No insurance	48.0 (27.5-69.1)	64.7 (45.1-80.4)
Political party affiliation		
Republican	54.6 (47.7-61.3)	43.0 (36.7-49.6)
Democrat	83.2 (77.0-88.0)	81.7 (75.7-86.5)
Independent^b^	66.7 (60.1-72.7)	63.8 (57.4-69.8)

Abbreviations: E, excellent; F, fair; G, good; P, poor; VG, very good.

^a^ Percentages correspond to those answering likely or very likely to vote for the candidate, in response to each vignette.

^b^ Independent includes respondents who selected independent or something else.

## Discussion

In this nationally representative survey study of US registered voters, we found majority support for candidates who would strongly endorse policies that are associated with child health across many topics. We also found that, even among policies with broad support, voter enthusiasm for candidates regarding child policy issues varied significantly within key demographic characteristics, such as gender, age, presence of children younger than 18 years in the household, and political party affiliation. Together, these findings suggest that candidates may wish to emphasize policy issues that are associated with child health more strongly in their campaigns for 2 main reasons: (1) these issues appeal to a majority of voters overall, and (2) simultaneously, there are evident differences in voter support associated with key voter characteristics (gender, political party) that are central to partisan politics in the current political environment.

### Medicaid

We found that two-thirds of registered voters would favor candidates who strongly support “offering Medicaid coverage through the federal government so that coverage is the same for everyone regardless of the state they live in.” Medicaid and the Child Health Insurance Program provide health insurance coverage for nearly 38 million US children, including children in households with a low income and those in foster care.^41^ Both programs are jointly financed by federal and state governments and administered by state governments. Medicaid is financed using a formula that considers a state’s per capita income, resulting in states with lower incomes receiving proportionally more federal funds and all states receiving at least 50% federal support.^42^ Despite this cost-sharing arrangement, Medicaid is commonly one of the largest annual state expenditures, making the program vulnerable, especially during economic downturns.^43,44^

State-level budget pressures from Medicaid have contributed to state-to-state variation in benefits and procedures. For example, after the COVID-19 public health emergency ended, states were again allowed to redetermine if Medicaid recipients met income eligibility requirements. As of May 14, 2024, more than 5 million children had lost Medicaid coverage,^45^ with substantial variation by state.^46^ While federalizing Medicaid is not a new idea, having been proposed in the early 2000s by former West Virginia Senator Jay Rockefeller,^47^ it recently garnered renewed attention after the American Academy of Pediatrics released a policy statement providing support again.^5^ The results of this study suggest that most voters in 2024 support this policy change.

### Refundable Child Tax Credit

The refundable child tax credit enacted as part of COVID-19 pandemic relief in 2021 was followed by immediate improvements in household income for families with children younger than 18 years.^6^ When the program was allowed to lapse, the reverse association with household income was immediate, jeopardizing children’s and families’ health and well-being.^7^ In this study, we found majority support for restoring the refundable child tax credit, which was enhanced in the survey-based experiment by framing the beneficiaries of this policy as “hard-working” vs “low-income” in randomly assigned vignettes. This framing was informed by a comparison of mass media messages about the refundable child tax credit,^19,20^ which suggests that media content may serve as a source for effective framing of health advocacy.

### Broad Support for Policies Associated With Child Health

The interconnectedness of health and social policy with child health is well-established^48,49^ and appeared to resonate with most voters in this study. Perhaps the most striking finding was the consistent majority support for candidates who would strongly support health and social policies affecting children. The highest, most uniform support was for ERPOs, which echoed recent surveys of adults living in states that had enacted ERPO laws that showed approximately three-quarters of respondents had positive perceptions of these “red flag laws,” with only small differences by political party.^50,51^ Broad support of gun violence prevention methods and legislative efforts resulted in the national Bipartisan Safer Communities Act of 2022, which contains several provisions for increased gun violence prevention, including allocation of federal resources for states to enact ERPOs.^52^

The findings of this study suggest that the lack of child-focused policy themes in political campaigns^1,2,3^ likely reflects underrecognition of how voters broadly favor child-focused campaign priorities. While our study found that voter support for child-focused policies was lower among Republicans than Independents and Democrats, there were popular policies among voters in each political party. This suggests that candidates whose principal focus is partisan politics may still find themes in child health and social policy that can be accommodated in their campaigns.

### Limitations

The results of this study should be interpreted in the context of certain limitations. First, while our sample was weighted to be representative of registered voters in the US, it is possible that some groups may not be fully represented due to sampling bias. To mitigate this risk, the panel used multiple methods to recruit a diverse sample, including providing internet-ready devices to those without a reliable connection. Second, online data collection may have biased our sample toward those who spend more time using the internet. This potential limitation is countered by the limitations of other modes of survey research, which may provide more biased samples (eg, nonweighted online panels, telephone polls). Third, we included many health and social policies to conduct our study, which reflect only a subset of all possible themes. Support for specific policies not included in this study would merit a similar research design. Fourth, as is common among surveys, we cannot exclude the possibility of response bias, including acquiescence bias, in which respondents may tend to have a positive response. The extent to which such bias may have occurred across study participants or across survey items cannot be ascertained. Fifth, as our survey was fielded in English and Spanish, non-English and non-Spanish speakers are not represented in our study findings. Sixth, voters expressed support for candidates who strongly support a range of child-focused policies, but our survey did not permit direct measurements of voters’ priorities for these specific policies within the broader context of other policies.

## Conclusions

The results of this survey study suggest that at the national level, most voters favor candidates who strongly support policies that are associated with child health. Voter support differs substantively by gender and political party affiliation and may be associated with framing choices in messaging about policy opportunities.

## Notes

Supplement 1. eAppendix. Survey Instrument eTable 1. Voter Support for Ensuring Consistent Medicaid Coverage for Children Across All States, Compared by Message Framing Presented in Randomly Assigned Vignettes eTable 2. Registered Voter Responses to Individual Child-Relevant Policies

Supplement 2. Data sharing statement

## References

[1] Benning TJ, Ashby GB, Chapp CB. Frequency and specificity of pediatric health policy discussions in political campaigns. JAMA Pediatr. 2020;174(8):795-796. doi:10.1001/jamapediatrics.2020.0932 32478807 PMC7265119

[2] Lambrew JM. Getting ready for health reform 2020: what past presidential campaigns can teach us. Accessed May 14, 2024. https://www.commonwealthfund.org/publications/fund-reports/2018/jun/getting-ready-health-reform-2020-presidential

[3] Galbraith AA, Carroll AE. Children’s health is too often ignored in elections—here is evidence to help change that. JAMA Pediatr. 2020;174(11):1026-1028. doi:10.1001/jamapediatrics.2020.3970 33016986

[4] Fowler EF, Moore ST, Floyd B, . Invoking identity? partisan polarization in discussions of race, racism, and gender in 2022 midterm advertising in the United States. J Health Polit Policy Law. 2024;49(3):505-537. doi:10.1215/03616878-11066296 37987197

[5] Kusma JD, Raphael JL, Perrin JM, Hudak ML; Committee on Child Health Financing. Medicaid and the Children’s Health Insurance Program: optimization to promote equity in child and young adult health. Pediatrics. 2023;152(5):e2023064088. doi:10.1542/peds.2023-064088 37860840

[6] Burns K, Fox L. The impact of the 2021 Expanded Child Tax Credit on child poverty. Accessed May 14, 2024. https://www.census.gov/library/working-papers/2022/demo/SEHSD-wp2022-24.html

[7] Sy S, Cuevas K. Child poverty increases sharply following expiration of expanded tax credit. Accessed May 14, 2024. https://www.pbs.org/newshour/show/child-poverty-increases-sharply-following-expiration-of-expanded-tax-credit

[8] Gitterman BA, Flanagan PJ, Cotton WH, ; Council on Community Pediatrics. Poverty and child health in the United States. Pediatrics. 2016;137(4):e20160339. doi:10.1542/peds.2016-0339 26962238

[9] Pallin R, Charbonneau A, Wintemute GJ, Kravitz-Wirtz N. California public opinion on health professionals talking with patients about firearms. Health Aff (Millwood). 2019;38(10):1744-1751. doi:10.1377/hlthaff.2019.00602 31589535

[10] Tipirneni R, Solway E, Malani P, . Health insurance affordability concerns and health care avoidance among US adults approaching retirement. JAMA Netw Open. 2020;3(2):e1920647. doi:10.1001/jamanetworkopen.2019.20647 32031644 PMC9578361

[11] Vogel EA, Ramo DE, Rubinstein ML, . Effects of social media on adolescents’ willingness and intention to use e-cigarettes: an experimental investigation. Nicotine Tob Res. 2021;23(4):694-701. 31912147 10.1093/ntr/ntaa003 PMC7976937

[12] Dunietz GL, Matos-Moreno A, Singer DC, Davis MM, O’Brien LM, Chervin RD. Later school start times: what informs parent support or opposition? J Clin Sleep Med. 2017;13(7):889-897. 10.5664/jcsm.6660 PMC5482580 28558863

[13] Freed GL, Davis MM, Singer DC, . Variation in generational perceptions of child health and well-being. Acad Pediatr. 2018;18(4):384-389. doi:10.1016/j.acap.2017.09.004 28919574

[14] Zamarripa A, Clark SJ, Rogers AJ, Wang-Flores H, Stanley RM. Pediatric concussion management in the emergency department: a national survey of parents. J Pediatr. 2017;181:229-234. doi:10.1016/j.jpeds.2016.10.071 27863850

[15] Patrick SW, Henkhaus LE, Zickafoose JS, . Well-being of parents and children during the COVID-19 pandemic: a national survey. Pediatrics. 2020;146(4):e2020016824. doi:10.1542/peds.2020-016824 32709738

[16] Heffernan ME, Bendelow A, Kociolek LK, Smith TL, Menker CG, Davis MM. Targeted vaccine messaging to promote COVID-19 vaccines for children and youth. Pediatrics. 2023;151(6):e2022059191. doi:10.1542/peds.2022-059191 37144291

[17] Gollust SE, Nelson KL, Purtle J. Selecting evidence to frame the consequences of adverse childhood experiences: testing effects on public support for policy action, multi-sector responsibility, and stigma. Prev Med. 2022;154:106912. doi:10.1016/j.ypmed.2021.106912 34921834

[18] Bruine de Bruin W, Wallin A, Parker AM, Strough J, Hanmer J. Effects of anti- versus pro-vaccine narratives on responses by recipients varying in numeracy: a cross-sectional survey-based experiment. Med Decis Making. 2017;37(8):860-870. doi:10.1177/0272989X17704858 28474962 PMC5623596

[19] Elkind E. House passes $78 billion tax bill expanding child tax credit and boosting US manufacturing. Accessed May 14, 2024. https://www.foxnews.com/politics/house-passes-78-billion-tax-bill-expanding-child-tax-credit-boosting-us-manufacturing

[20] Foran C, Luhby T, Wilson K, Talbot H. House passes bipartisan tax bill that expands child tax credit. Accessed May 14, 2024. https://www.cnn.com/2024/01/31/politics/house-vote-tax-bill-child-tax-credit/index.html

[21] Blau DM. The effects of universal preschool on child and adult outcomes: a review of recent evidence from Europe with implications for the United States. Early Child Res Q. 2021;55:52-63. doi:10.1016/j.ecresq.2020.10.009

[22] Chor E, Andresen ME, Kalil A. The impact of universal prekindergarten on family behavior and child outcomes. Econ Educ Rev. 2016;55:168-181. doi:10.1016/j.econedurev.2016.10.002

[23] Hotz VJ, Wiswall M. Child care and child care policy: existing policies, their effects, and reforms. Ann Am Acad Pol Soc Sci. 2019;686(1):310-338. doi:10.1177/0002716219884078

[24] Localio AM, Knox MA, Basu A, Lindman T, Walkinshaw LP, Jones-Smith JC. Universal free school meals policy and childhood obesity. Pediatrics. 2024;153(4):e2023063749. doi:10.1542/peds.2023-063749 38495019 PMC10979297

[25] Cohen JFW, Hecht AA, McLoughlin GM, Turner L, Schwartz MB. Universal school meals and associations with student participation, attendance, academic performance, diet quality, food security, and body mass index: a systematic review. Nutrients. 2021;13(3):911. doi:10.3390/nu13030911 33799780 PMC8000006

[26] Marcus M, Yewell KG. The effect of free school meals on household food purchases: evidence from the community eligibility provision. J Health Econ. 2022;84:102646. doi:10.1016/j.jhealeco.2022.102646 35792362

[27] Collins AM, Klerman JA, Briefel R, . A summer nutrition benefit pilot program and low-income children’s food security. Pediatrics. 2018;141(4):e20171657. doi:10.1542/peds.2017-1657 29592869

[28] Kingshipp BJ, Scinto-Madonich S, Bahnfleth C, Cole NC, Butera G, Spahn J. USDA-Funded Summer Feeding Programs and Key Child Health Outcomes of Public Health Importance: A Rapid Review. USDA Nutrition Evidence Systematic Review; 2022. 38085841

[29] Bullinger LR. The effect of paid family leave on infant and parental health in the United States. J Health Econ. 2019;66:101-116. doi:10.1016/j.jhealeco.2019.05.006 31150953

[30] Jou J, Kozhimannil KB, Abraham JM, Blewett LA, McGovern PM. Paid maternity leave in the United States: associations with maternal and infant health. Matern Child Health J. 2018;22(2):216-225. doi:10.1007/s10995-017-2393-x 29098488

[31] Miller ER, Hudak ML. Medicaid and newborn care: challenges and opportunities. J Perinatol. 2023;43(8):1072-1078. doi:10.1038/s41372-023-01714-4 37438483

[32] Lee LK, Fleegler EW, Goyal MK, . Firearm-related injuries and deaths in children and youth. Pediatrics. 2022;150(6):e2022060071. doi:10.1542/peds.2022-060071 36207778

[33] Cornell D, Maeng J. Statewide implementation of Threat Assessment in Virginia K-12 Schools. Contemp Sch Psychol. 2018;22(2):116-124. doi:10.1007/s40688-017-0146-x

[34] Rajan S, Reeping PM, Ladhani Z, Vasudevan LM, Branas CC. Gun violence in K-12 schools in the United States: moving towards a preventive (versus reactive) framework. Prev Med. 2022;165(Pt A):107280. doi:10.1016/j.ypmed.2022.107280 36183796 PMC12755125

[35] Monuteaux MC, Azrael D, Miller M. Association of increased safe household firearm storage with firearm suicide and unintentional death among US youths. JAMA Pediatr. 2019;173(7):657-662. doi:10.1001/jamapediatrics.2019.1078 31081861 PMC6515586

[36] Brantley E, Ku L. Continuous eligibility for Medicaid associated with improved child health outcomes. Med Care Res Rev. 2022;79(3):404-413. doi:10.1177/10775587211021172 34525877

[37] Census US. Complete technical documentation—current population survey. Accessed May 16, 2024. https://www.census.gov/programs-surveys/cps/technical-documentation/complete.html

[38] Census US. American community survey. Accessed May 16, 2024. https://www.census.gov/programs-surveys/acs

[39] Lumley T. Analysis of complex survey samples. J Stat Softw. 2004;9:1-19. doi:10.18637/jss.v009.i08

[40] Callegaro M, DiSogra C. Computing response metrics for online panels. Public Opin Q. 2008;72(5):1008-1032. doi:10.1093/poq/nfn065

[41] US Centers for Medicare & Medicaid Services. January 2024 Medicaid & CHIP Enrollment Data Highlights. Accessed May 14, 2024. https://www.medicaid.gov/medicaid/program-information/medicaid-and-chip-enrollment-data/report-highlights/index.html

[42] Patrick SW, Davis MM. Reformulating the federal match as a key to the sustainability of Medicaid. JAMA Pediatr. 2013;167(3):218-220. doi:10.1001/jamapediatrics.2013.1075 23318607

[43] Patrick SW, Choi H, Davis MM. Increase in federal match associated with significant gains in coverage for children through Medicaid and CHIP. Health Aff (Millwood). 2012;31(8):1796-1802. doi:10.1377/hlthaff.2011.0988 22869658

[44] KFF. Medicaid expenditures as a percent of total state expenditures by fund. Accessed May 14, 2024. https://www.kff.org/medicaid/state-indicator/medicaid-expenditures-as-a-percent-of-total-state-expenditures-by-fund/

[45] Georgetown University McCourt School of Public Policy Center for Children and Families. Unwinding continuous coverage. Accessed May 14, 2024. https://ccf.georgetown.edu/subtopic/unwinding-phe/

[46] Georgetown University McCourt School of Public Policy Center for Children and Families. What is happening with Medicaid renewals in each state? Accessed May 14, 2024. https://ccf.georgetown.edu/2023/07/14/whats-happening-with-medicaid-renewals/

[47] Tharp M. MediKids bill introduced in new Congress. AAP News. 2003;22(5):196-196. doi:10.1542/22.5.196a

[48] Reynolds MM, Homan PA. Income support policy packages and birth outcomes in U.S. states: an ecological analysis. Popul Res Policy Rev. 2023;42(4):73. doi:10.1007/s11113-023-09797-9 38213513 PMC10783327

[49] Galbraith A, Flanagin A, Carroll AE, . JAMA Network call for papers on health and the 2024 US election. JAMA. 2023;330(10):923-924. doi:10.1001/jama.2023.14719

[50] Ad Council Research Institute. RPOs: Understanding Public Knowledge & Attitudes Toward Extreme Risk Protection Orders. Ad Council Research Institute; 2023.

[51] APM Research Lab. Americans’ views on key gun policies: part one: opinions on “red flag” laws. Accessed May 15, 2024. https://www.apmresearchlab.org/gun-survey-red-flag

[52] US Congress. S.2938–Bipartisan Safer Communities Act. Accessed May 14, 2024. https://www.congress.gov/bill/117th-congress/senate-bill/2938/text

