# Supplementary material for: Voter Support for Policies Associated With Child Health as National Campaign Priorities
Source: JAMA Health Forum. 2024 Sep 27;5(9):e243305. doi: 10.1001/jamahealthforum.2024.3305 (PMC11437383; doi:10.1001/jamahealthforum.2024.3305)
Supplement: Supplement 1. — eAppendix. Survey Instrument eTable 1. Voter Support for Ensuring Consistent Medicaid Coverage for Children Across All States, Compared by Message Framing Presented in Randomly Assigned Vignettes eTable 2. Registered Voter Responses to Individual Child-Relevant Policies [file jamahealthforum-e243305-s001.pdf]

Supplemental Online Content

Patrick SW, Loch SF, McNeer E, Davis MM. Voter support for policies associated with child health as national campaign priorities. *JAMA Health Forum*. 2024;5(9):e243305.
doi:10.1001/jamahealthforum.2024.3305

eAppendix. Survey Instrument
eTable 1. Voter Support for Ensuring Consistent Medicaid Coverage for Children Across All States, Compared by Message Framing Presented in Randomly Assigned Vignettes
eTable 2. Registered Voter Responses to Individual Child-Relevant Policies

This supplemental material has been provided by the authors to give readers additional information about their work.

© 2024 Patrick SW et al. *JAMA Health Forum.*

eAppendix

Survey Instrument

Sample Variables

- KP standard demographics
- XVOTEREG_NOW: Whether currently registered to vote (-2=Not asked, -1=Refused, 1=Yes, I am registered to vote, 3=No, I am not registered to vote, 4=Not sure, 5=No, I am not eligible to vote)
- XPARENT: Parental Status of Children 0-17 (1=parents of children 0-17 YO; 2=not parents of children 0-17 YO; 9=missing data)
- XSPANISH
- XACSLANG
- XURBANICITY
- XPARTY4
- XPOLICY: Sample (1=Gen Pop, 2=Parent Augment)

Phase. [S]:
1. Pretest [n20]
2. Main [n2000]

Standard Question Type Descriptions

Standard question types include:

- *S = Single Select: Allows respondents to select one answer in a list of options.*
- *Grid (including options for banked or accordion grids)*
- *S (Optional: Banked/Accordion) Grid: Allows respondents to select one answer in a 2-dimensional grid layout.*
- *M (Optional: Banked/Accordion) Grid: Allows respondents to select multiple answers in a 2-dimensional grid layout.*
- *N = Number: Allows respondents to enter a numeric response in an open-ended answer field (specify valid range or number of digits, e.g., up to three digits for age, five numbers for zip code)*
- *DISP = Display/Descriptive Content: Displays text and/or multimedia elements to respondents without requiring interaction.*

Create DOV_Order, where 1 =-'top-to-bottom' and 2 = 'bottom-to-top'
Randomly assign the DOV_Order to be used throughout survey to a single respondent

© 2024 Patrick SW et al. *JAMA Health Forum.*

SCREENER

Base: All respondents

INTRO [DISP]

Like all surveys, this survey may include some personal questions. This survey may include some questions about your thoughts on social, political, and health issues. As a reminder, your responses are only ever used for research purposes and will remain anonymous – results are reported only for groups, not for individuals. You can choose not to answer any question. Answering the questions means that you accept us collecting the data.

BASE: ALL RESPONDENTS

REGVOTE [S]

To start, are you currently registered to vote in the U.S.?

1. Yes, I am registered to vote
2. No, I am not registered to vote
3. Not sure
4. No, I am not eligible to vote

IF REGVOTE IS 2, 3, 4 OR REFUSED, THANK AND TERMINATE.

BASE: ALL RESPONDENTS

PARENT [N]

Next, how many children age 18 or younger are living in your household? Please only include the children for whom you are the parent. If you have no children age of 18 or younger in your household, please answer “0”.

INSERT NUMBER BOX RANGE 0-10

______ child/children from 0 to 18 years old

IF PARENT=0 OR REFUSED AND XPOLICY=2 THANK AND TERMINATE.

IF XPOLICY = 1 and REGVOTE = 1, QFLAG = 1.
IF XPOLICY = 1 and REGVOTE = 2 or REFUSED, QFLAG = 2.
IF XPOLICY = 2 and PARENT > 0, QFLAG = 1.
IF XPOLICY = 2 and PARENT = 0 or REFUSED, QFLAG = 2.

© 2024 Patrick SW et al. *JAMA Health Forum.*

MAIN QUESTIONNAIRE
SURVEY-BASED EXPERIMENT

CREATE DOV_VIGNETTE3, AND RANDOMLY ASSIGN PARTICIPANTS (LEAST FILL) TO ONE OF THE THREE FOLLOWING VIGNETTES

Base: All Respondents

INTRO [DISP]

Medicare and Medicaid are two government health insurance programs in the United States. Both programs help Americans pay for healthcare.

Medicare is available to seniors 65 years old and older, no matter what state they live in. Only the federal government pays for Medicare.

Medicaid is different: a child can be eligible for Medicaid coverage in one state but not in another state. That is true even if the child has health problems.

That's because states make their own decisions about which children are eligible for Medicaid, based on several criteria. States are allowed to be different from each other because they share in paying for Medicaid, along with the federal government.

[NEW SCREEN]
EQUITY frame, DISPLAY:

In other words: seniors are treated equally across states under Medicare. But children are not treated equally at all in Medicaid programs across those same states. As a result, children in states with less generous Medicaid coverage are more likely to be uninsured, and less likely to less access to health care.

Some experts have called for a new national program that would ensure that all children have equal chance of having Medicaid coverage, no matter what state they live in – just like Medicare for seniors.

,FAIRNESS frame, DISPLAY:

In other words: seniors are treated the same across states under Medicare, but children are not. This is plainly unfair for kids who are not eligible for Medicaid, just based on what state they live in. As a result, children in states with less generous Medicaid coverage are more likely to be uninsured, and less likely to less access to health care.

Some experts have called for a new national program that would ensure that all children have a fair chance of having Medicaid coverage, no matter what state they live in – just like Medicare for seniors.

LOSS frame, DISPLAY:

In other words: all seniors benefit from Medicare, no matter what state they live. But children in some states simply lose out on Medicaid coverage just because of where they live. As a result, children in states with less generous Medicaid coverage are more likely to be uninsured, and less likely to less access to health care.

Some experts have called for a new national program that would ensure that no children lose out on Medicaid coverage, no matter what state they live in – just like Medicare for seniors.

Base: All respondents

[DISPLAY ON SAME SCREEN AS SELECTED DOV_VIGNETTE]

Would you favor or oppose a new national program that would ensure that Medicaid coverage is available to all children in all states?

ROTATE 1-5, 5-1, same DOV_order

1. Strongly oppose
2. Oppose
3. Neither favor nor oppose

© 2024 Patrick SW et al. *JAMA Health Forum.*

4. Favor
5. Strongly favor

© 2024 Patrick SW et al. *JAMA Health Forum.*

MAIN QUESTIONNAIRE
SURVEY-BASED EXPERIMENT

CREATE DOV_VIGNETTE4, AND RANDOMLY ASSIGN PARTICIPANTS (LEAST FILL) TO ONE OF THE TWO FOLLOWING VIGNETTES

Base: All respondents

HARDWORKING FAMILIES language, DISPLAY:

Since 1997, families with children have benefited from the child tax credit, which helped reduce their overall taxes. In 2021, the child tax credit became 'refundable.' This meant that even families that were required to pay little or no tax received direct payments for children in their households.

The refundable child tax credit was designed to provide more immediate financial support to hardworking families with children. It worked: child poverty rates decreased, and the well-being of children improved across the United States.

However, Congress reversed the refundable child tax credit in 2022. As a result, child poverty levels returned to pre-2021 levels. Congress is now considering whether to reinstate refundable child tax credits for hardworking families.

LOW-INCOME FAMILIES language, DISPLAY

Since 1997, families with children have benefited from the child tax credit, which helped reduce their overall taxes. In 2021, the child tax credit became ‘refundable.’ This meant that even families that were required to pay little or no tax received direct payments for children in their households.

The refundable child tax credit was designed to provide more immediate financial support to low-income families with children. It worked: child poverty rates decreased, and the well-being of children improved across the United States.

However, Congress reversed the refundable child tax credit in 2022. As a result, child poverty levels returned to pre-2021 levels. Congress is now considering whether to reinstate refundable child tax credits for low-income families.

DISPLAY TO ALL:

Would you favor or oppose Congress making the child tax credit refundable again?

ROTATE 1-5, 5-1, same DOV_order

1. Strongly oppose
2. Oppose
3. Neither favor nor oppose
4. Favor
5. Strongly favor

© 2024 Patrick SW et al. *JAMA Health Forum.*

MAIN QUESTIONNAIRE
VOTING PREFERENCES FOR CANDIDATES BASED ON SPECIFIC HEALTHCARE ISSUES

PROGRAMMER: RANDOMIZE ORDER OF QUESTIONS. SPLIT QUESTIONS ACROSS THREE SCREENS WITH 4 STATEMENTS ON ONE SCREEN AND THREE EACH ON OTHER TWO SCREENS,

Base: All respondents

For each of the following types of programs, please tell us how likely or unlikely you would be to vote for a candidate who said they would strongly support national funding for that program.

Base: All respondents

And for each of the following types of programs, please tell us how likely or unlikely you would be to vote for a candidate who said they would strongly support national funding for that program.

Base: All respondents

And, for each of the following types of programs, please tell us how likely or unlikely you would be to vote for a candidate who said they would strongly support national funding for that program.

Base: All respondents

[ACCORDION GRID, S]

Statements in rows; RANDOMIZE

Q. Universal free preschool for 3- and 4-year-olds.
Q. Expanding the availability of high-quality, affordable childcare.
Q. Expanding free school meals for school-aged children.
Q. Establishing summer nutrition program that provides money to families of school-aged children to purchase food when school is not in session.
Q. Requiring paid parental leave for the birth or adoption of a child.
Q. Offering Medicaid coverage through the federal government so that coverage is the same for everyone regardless of the state they live in.
Q. Temporarily preventing someone in mental health crisis from accessing firearms.
Q. Establishing threat assessment and identification programs for schools to prevent active shooter events.
Q. Providing safe storage (trigger locks, safes) for all new firearm purchases and requiring that unattended firearms are stored securely.
Q. Preventing states from removing children less than 6 years old from Medicaid.

Responses in columns; ROTATE 1-5, 5-1 same DOV_order

1. Would DEFINITELY NOT vote for the candidate
2. Would LIKELY NOT vote for the candidate
3. Not sure
4. Would LIKELY vote for the candidate
5. Would DEFINITELY vote for the candidate

© 2024 Patrick SW et al. *JAMA Health Forum.*

MAIN QUESTIONNAIRE
DEMOGRAPHIC QUESTIONS

Base: All respondents

The following questions are for classification purposes only. Please be assured that your responses will be aggregated with those of other participants to this survey.

Base: All respondents

INSURE [M]

Below is a list of different kinds of health insurance. Are you covered by any of the following types of health insurance or health coverage plans?

Please do not include Medicare supplements or specialty insurance such as dental or long-term care.

Select all answers that apply.

1. Insurance through a current or former employer or union (your employer or another family member's). These plans may be with companies such as Aetna, UnitedHealth, Blue Cross Blue Shield, etc.
2. Medicare, for people 65 and older, or people with certain disabilities
3. Medicaid, Medical Assistance, or any kind of government-assistance plan for those with low incomes or a disability
4. Insurance purchased directly from an insurance company (including "Obamacare" or a state exchange).
8. TRICARE or other military health care
5. Veteran's Affairs (VA), Department of Defense, or other military programs
9. Indian Health Service
6. Any other type of health insurance or health coverage plan please specify [O]
7. I don't have health insurance [S]

Base: All respondents

HEALTH [S]

In general, would you say your physical health is...?

1. Excellent
2. Very good
3. Good
4. Fair
5. Poor

© 2024 Patrick SW et al. *JAMA Health Forum.*

Base: All respondents

KIDHEALTH [S]

How would you describe the health of [*IF PARENT = 1, INSERT:* the; *IF PARENT > 1, INSERT:* the oldest] child age 0-17 living in your household?

1. Excellent
2. Very good
3. Good
4. Fair
5. Poor

© 2024 Patrick SW et al. *JAMA Health Forum.*

eTable 1. Voter Support for Ensuring Consistent Medicaid Coverage for Children Across All States, Compared by Message Framing Presented in Randomly Assigned Vignettes.

	**Equity**<br>N = 674<br>% (95%CI)	**Fairness**<br>N = 670<br>% (95%CI)	**Loss Avoidance**<br>N = 661<br>% (95%CI)
Gender			
Male	66.2% (59.6-72.2)	63.0% (56.4-69.3)	63.3% (56.5-69.7)
Female	74.9% (68.8-80.1)	74.7% (68.8-79.8)	78.0% (71.8-83.1)
Age			
18-29	74.0% (59.5-84.7)	66.7% (51.6-79.0)	75.9% (60.2-86.7)
30-44	73.4% (65.2-80.3)	76.7% (68.8-83.1)	70.8% (62.4-78.0)
45-59	68.4% (59.5-76.2)	66.0% (56.9-74.1)	71.2% (61.8-79.1)
60+	67.7% (60.2-74.5)	66.5% (59.2-73.0)	68.4% (61.2-74.9)
Parental Status			
Parent	74.8% (69.5-79.6)	69.5% (63.8-74.5)	72.4% (66.8-77.4)
Non-Parent	69.2% (63.7-74.3)	69.0% (63.5-74.0)	70.5% (64.6-75.7)
Urbanicity			
Urban	74.0% (66.8-80.0)	72.4% (65.0-78.8)	76.3% (68.1-82.9)
Suburban	69.1% (62.1-75.3)	69.5% (62.7-75.5)	67.7% (60.7-74.0)
Rural	67.1% (56.7-76.2)	61.3% (50.8-70.9)	69.5% (59.9-77.7)
Health Status - Adult			
Worse (F/P)	73.7% (61.5-83.1)	80.0% (68.3-88.2)	79.4% (67.4-87.8)
Better (E/VG/G)	69.9% (65.0-74.3)	66.8% (61.9-71.3)	69.1% (64.2-73.7)
Health Status - Child			
Worse (G/F/P)	71.3% (51.3-85.4)	63.3% (43.9-79.2)	84.5% (67.8-93.4)
Better (E/VG)	75.4% (69.8-80.2)	70.8% (64.9-76.0)	70.5% (64.4-75.9)
Insurance Type			
Public	71.2% (62.5-78.5)	67.4% (58.7-75.0)	67.8% (58.9-75.6)
Private	70.8% (65.4-75.7)	71.3% (66.0-76.1)	71.5% (65.8-76.6)
Other	100% (100-100)	45.6% (7.5-89.6)	53.0% (6.5-94.8)
No Insurance	59.7% (36.2-79.5)	50.4% (22.1-78.5)	82.1% (60.9-93.2)
Political Party Affiliation			
Republican	46.0% (38.3-53.9)	54.9% (46.5-63.1)	50.2% (42.0-58.5)
Democrat	90.9% (84.3-94.9)	87.2% (80.9-91.7)	87.6% (79.5-92.8)
Independent[a]	71.8% (64.0-78.6)	59.1% (51.1-66.8)	72.6% (65.1-79.0)

Percentages correspond to those answering "likely" or "very likely" in response to each vignette
[a]Independent includes respondents who selected independent or “something else” when asked for their political party affiliation.

© 2024 Patrick SW et al. *JAMA Health Forum.*

eTable 2. Registered Voter Responses to Individual Child-Relevant Policies.

		% (95% CI)
**Universal Free Preschool**	Would DEFINITELY NOT vote for the candidate	6.6% (5.4-8.1)
	Would LIKELY NOT vote for the candidate	8.5% (7.2-10.0)
	Not sure	23.3% (21.1-25.7)
	Would LIKELY vote for the candidate	32.9% (30.4-35.5)
	Would DEFINITELY vote for the candidate	28.7% (26.3-31.2)
**Expanded Childcare**	Would DEFINITELY NOT vote for the candidate	3.8% (2.9-5.0)
	Would LIKELY NOT vote for the candidate	5.5% (4.4-6.9)
	Not sure	21.0% (18.9-23.3)
	Would LIKELY vote for the candidate	41.2% (38.5-43.9)
	Would DEFINITELY vote for the candidate	28.4% (26.1-30.9)
**Free School Meals**	Would DEFINITELY NOT vote for the candidate	4.8% (3.8-6.0)
	Would LIKELY NOT vote for the candidate	9.7% (8.3-11.3)
	Not sure	20.0% (17.9-22.2)
	Would LIKELY vote for the candidate	33.5% (31.0-36.2)
	Would DEFINITELY vote for the candidate	32.0% (29.5-34.6)
**Summer Nutrition Programs**	Would DEFINITELY NOT vote for the candidate	5.4% (4.4-6.7)
	Would LIKELY NOT vote for the candidate	11.1% (9.5-12.9)
	Not sure	25.6% (23.3-28.0)
	Would LIKELY vote for the candidate	34.9% (32.3-37.5)
	Would DEFINITELY vote for the candidate	23.0% (20.8-25.4)
**Paid Parental Leave**	Would DEFINITELY NOT vote for the candidate	5.3% (4.2-6.6)
	Would LIKELY NOT vote for the candidate	7.9% (6.6-9.4)
	Not sure	21.3% (19.2-23.6)
	Would LIKELY vote for the candidate	37.5% (34.9-40.2)
	Would DEFINITELY vote for the candidate	28.0% (25.6-30.5)

© 2024 Patrick SW et al. *JAMA Health Forum.*

**Federalized Medicaid**	Would DEFINITELY NOT vote for the candidate	5.6% (4.5-7.0)
	Would LIKELY NOT vote for the candidate	7.7% (6.4-9.1)
	Not sure	20.7% (18.6-23.0)
	Would LIKELY vote for the candidate	35.1% (32.5-37.7)
	Would DEFINITELY vote for the candidate	30.9% (28.5-33.5)
**Extreme Risk Protection Order**	Would DEFINITELY NOT vote for the candidate	2.6% (1.9-3.5)
	Would LIKELY NOT vote for the candidate	2.6% (1.9-3.6)
	Not sure	15.3% (13.3-17.4)
	Would LIKELY vote for the candidate	32.4% (30.0-35.0)
	Would DEFINITELY vote for the candidate	47.1% (44.4-49.8)
**School Threat Assessment**	Would DEFINITELY NOT vote for the candidate	2.1% (1.4-3.1)
	Would LIKELY NOT vote for the candidate	3.2% (2.4-4.2)
	Not sure	21.6% (19.5-24.0)
	Would LIKELY vote for the candidate	39.0% (36.4-41.7)
	Would DEFINITELY vote for the candidate	34.1% (31.5-36.7)
**Safe Firearm Storage and Enforcement**	Would DEFINITELY NOT vote for the candidate	6.2% (5.1-7.6)
	Would LIKELY NOT vote for the candidate	8.5% (7.1-10.2)
	Not sure	22.3% (20.1-24.6)
	Would LIKELY vote for the candidate	32.3% (29.8-34.9)
	Would DEFINITELY vote for the candidate	30.6% (28.2-33.2)
**Prevent Medicaid Disenrollment for Children <6 Years Old**	Would DEFINITELY NOT vote for the candidate	4.3% (3.4-5.5)
	Would LIKELY NOT vote for the candidate	5.6% (4.5-6.9)
	Not sure	28.2% (25.8-30.7)
	Would LIKELY vote for the candidate	33.5% (30.9-36.1)
	Would DEFINITELY vote for the candidate	28.4% (26.0-30.9)
**Child Tax Credit**	Strongly oppose	5.8% (4.7-7.2)
	Oppose	7.8% (6.5-9.3)
	Neither favor nor oppose	19.8% (17.6-22.1)
	Favor	34.2% (31.7-36.8)
	Strongly favor	32.4% (29.9-35.0)

© 2024 Patrick SW et al. *JAMA Health Forum.*

© 2024 Patrick SW et al. *JAMA Health Forum.*
