# Supplementary material for: Voter Support for Policies Associated With Child Health as National Campaign Priorities
Source: JAMA Health Forum. 2024 Sep 27;5(9):e243305. doi: 10.1001/jamahealthforum.2024.3305 (PMC11437383; doi:10.1001/jamahealthforum.2024.3305)
Supplement: Supplement 2. — Data sharing statement [file jamahealthforum-e243305-s002.pdf]

Data Sharing Statement

Patrick. Voter Support for Policies Associated With Child Health as National Campaign Priorities. *JAMA Health Forum*. Published September 27, 2024.
doi:10.1001/jamahealthforum.2024.3305

Data
Data available: Yes
Data types: Deidentified participant data, Data dictionary
How to access data: stephen.patrick@emory.edu
When available: beginning date: 06-01-2025

Supporting Documents
Document types: Statistical/analytic code
How to access documents: stephen.patrick@emory.edu
When available: beginning date: 06-01-2025

Additional Information
Who can access the data: researchers whose proposed use of the data has been approved
Types of analyses: for any purpose
Mechanisms of data availability: after approval
Any additional restrictions: none
